# Usefulness of Preoperative Oral Screening for Safe Anesthesia Management

**DOI:** 10.7759/cureus.98235

**Published:** 2025-12-01

**Authors:** Yoshiko Yamamura, Shiho Koroku, Yumena Morimoto, Yuki Sato, Junko Yamazaki, Seiji Ishikawa, Izumi Kawagoe, Shunsuke Namaki

**Affiliations:** 1 Department of Oral and Maxillofacial Surgery, Faculty of Medicine, Juntendo University Hospital, Tokyo, JPN; 2 Department of Anesthesiology and Pain Medicine, Faculty of Medicine, Juntendo University, Tokyo, JPN; 3 Department of Oral and Maxillofacial Surgery, Faculty of Medicine, Juntendo University, Tokyo, JPN

**Keywords:** general anesthesia, incident, oral screening, questionnaire survey, selective dental intervention system

## Abstract

Objective: Preoperative assessment plays a crucial role in ensuring patient safety and minimizing postoperative complications. To improve perioperative management, a preoperative outpatient clinic was established at our hospital for patients scheduled to undergo surgery or examination under anesthesia. Given the high number of patients requiring anesthesia in our hospital, it is not feasible for dentists to examine all the patients preoperatively. This study aimed to evaluate the effectiveness and challenges of a selective dental intervention system based on preoperative oral screening by dental hygienists for patients undergoing anesthesia. The primary outcome was the incidence of dental complications during anesthesia, and the secondary outcomes were the referral patterns for dental intervention and the anesthesiologists’ perceptions regarding the system.

Methods: We investigated and compared dental issues before (January 2012-October 2019) and after (November 2019-December 2024) establishing the preoperative outpatient dental screening clinic. Additionally, a questionnaire survey was conducted with anesthesiologists to determine their perceptions of the need for dental intervention.

Results: There were 32 cases of dental problems during anesthesia between January 2012 and December 2024. This number decreased after the establishment of the preoperative outpatient clinic in November 2019 (p=0.036). The questionnaire revealed that anesthesiologists recognized dental risks during anesthesia, particularly during intubation and extubation, based on notes from dental hygienists, which helped alert them to potential issues.

Conclusions: Providing dental interventions by dentists for all patients scheduled for anesthesia is not possible owing to human resource and time constraints. Although some issues persist, such as the inability to inspect diagnostic images and a lack of clarity regarding lesions within the jawbone, our findings suggest that preoperative oral screening plays an important role in ensuring safe anesthesia management.

## Introduction

Dental problems, such as tooth loss or fracture, during general anesthesia, which have been reported in 0.04%-12.1% of cases [1-4], are an extremely serious issue that can lead to lawsuits. We must be aware of this problem and work towards preventing its occurrence.

A preoperative outpatient clinic was established at our hospital in May 2019 for patients scheduled for anesthesia, with the aim of providing safe management and reducing postoperative complications. Preoperative oral screening began in November 2019. This clinic provides vital checks, medication guidance from pharmacists, an explanation of the hospital stay, a preoperative examination by anesthesiologists, and oral screening by dental hygienists for intervention by dentists. Prosthetic devices, dentures, and orthodontic appliances are checked; loose teeth, trismus, and temporomandibular joint symptoms are identified; oral hygiene is assessed; and the need for dental intervention is noted. If dental problems are recognized, the concerned patient is advised to undergo treatment at our department or their family dental clinic prior to undergoing surgery. As many patients require anesthesia management at our hospital (general anesthesia: approximately 11,000 cases/year), performing dental interventions for all patients is not feasible for dentists. We believe that preoperative oral screening by dental hygienists can improve the safety of patient management and prevent adverse complications. Therefore, the objective of this study was to evaluate the usefulness and challenges of a selective dental intervention system based on preoperative oral screening by dental hygienists. Specifically, we aimed (1) to determine whether the introduction of oral screening reduced the incidence of dental-related complications during general anesthesia, and (2) to assess referral patterns and anesthesiologists’ perceptions of the screening system. Although the number of dental incidents was small relative to the total number of anesthetic procedures, we considered that analyzing these cases could provide meaningful insights into the potential benefits and limitations of the system in a high-volume tertiary hospital setting.

## Materials and methods

Study design

**Figure 1:**
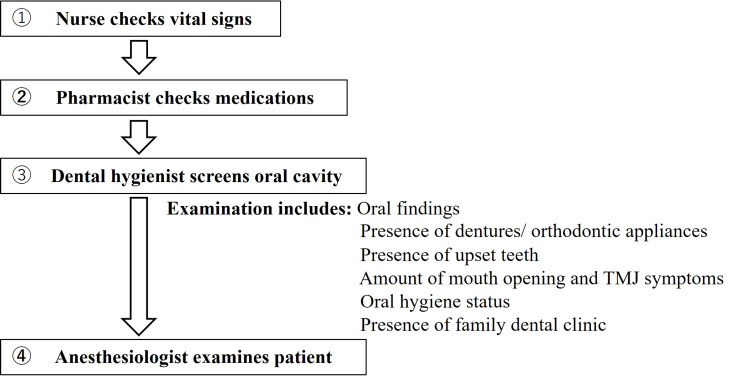
Preoperative outpatient flow TMJ: temporomandibular joint

The preoperative outpatient flow is shown in Figure 1. To evaluate the usefulness of the selective dental intervention system, we reviewed information on referrals from the preoperative outpatient clinic to our department from November 2019 (when the preoperative oral screening was started) to March 2024 and analyzed the nature of these requests. All referrals were consecutively included without exclusion criteria. The total number of anesthesia cases during the same period was obtained from the hospital anesthesia registry to serve as the denominator for calculating incident rates.

Searched for dental-related incidents

Hospital records were searched for dental-related incidents that occurred during anesthesia between January 2012 and December 2024 using the keywords “anesthesia,” “tooth,” “loss,” “deficiency,” and “fracture.” Filters were set to include only perioperative notes and anesthesia records. The number and details of reported dental problems during anesthesia were investigated. Thirty-two cases of dental-related incidents were reported during general anesthesia procedures conducted between January 2012 and December 2024. Since the implementation of oral screening in November 2019, we separated incidents into two categories: those that occurred before the establishment of oral screening (January 2012 to October 2019: 24 cases) and those that occurred after the establishment of the preoperative clinic (November 2019 to December 2024: eight cases).

Questionnaire survey

A questionnaire survey was conducted with anesthesiologists to determine (1) whether doctors in the Department of Anesthesiology and Pain Clinic felt that oral screening was useful, (2) whether dental problems were resolved through preoperative oral screening, and (3) whether or not a doctor can be alerted to tooth fractures or loss of teeth in patients during anesthesia management based on the dental hygienist's documentation of oral screening. The questionnaire was originally developed by the authors based on the study objectives and previous literature on preoperative oral management. The items were reviewed by two dental professionals to ensure clarity and relevance before distribution. The final instrument consisted of five close-ended questions with five-point Likert-scale responses (1 = strongly disagree to 5 = strongly agree). The full questionnaire is provided in Appendix A. Responses were anonymous and collected electronically.

Statistical analysis

The number of dental complications during general anesthesia was compared before and after the establishment of the preoperative outpatient clinic using the chi-square test (Social Survey Research Information Co., Ltd., Tokyo, Japan). A p-value of <0.05 was considered statistically significant.

## Results

Referral status and requests from the preoperative outpatient clinic to our department

**Figure 2:**
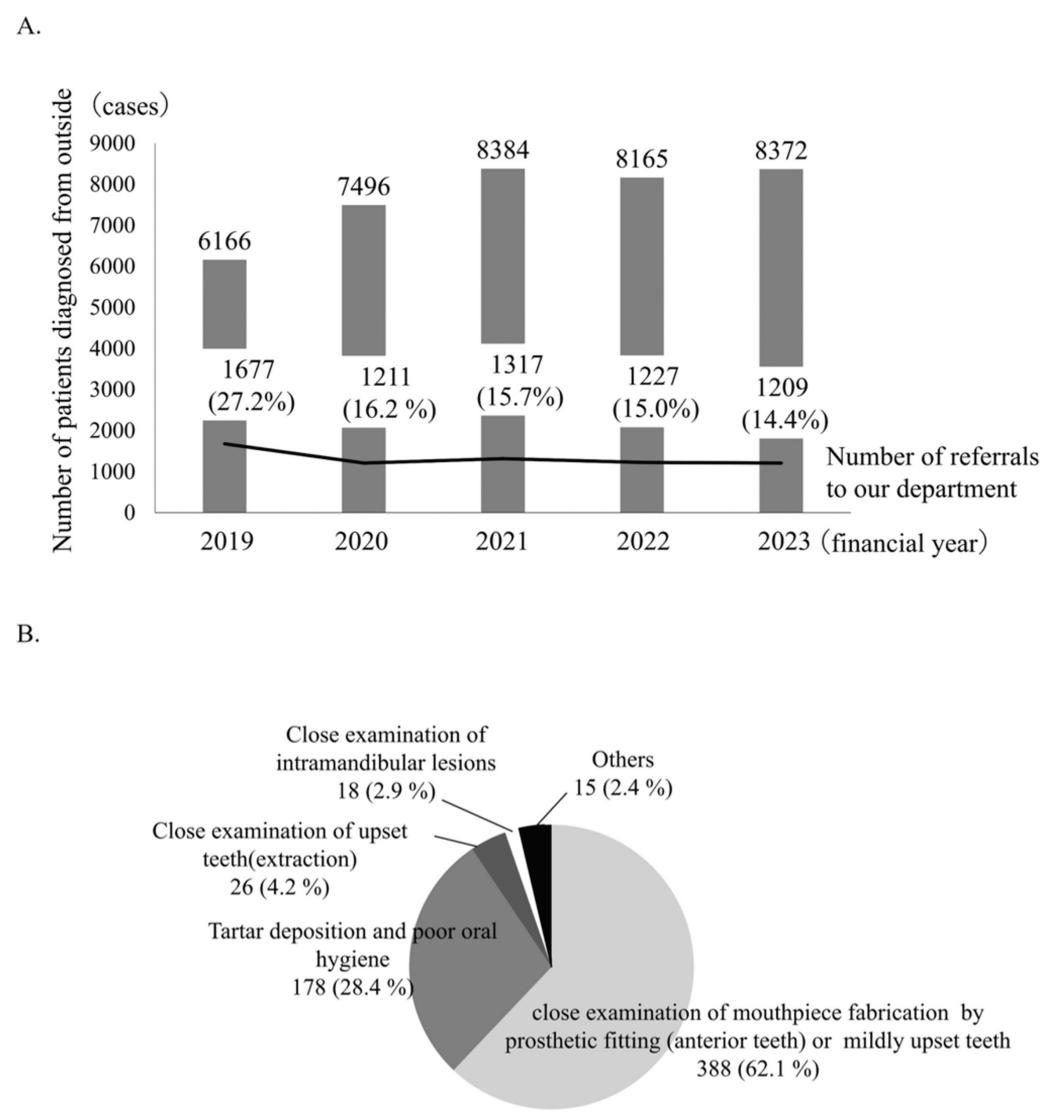
Referral status and requests from the preoperative outpatient clinic to our department (A) Status of referrals from the preoperative outpatient to our department; (B) Details of the request from the preoperative outpatient clinic to our department (10/2023-3/2024: 581 cases)

Approximately 15% of patients were referred to our department for oral examination requests from the preoperative outpatient clinic (Figure 2A). There were 581 requests from the preoperative outpatients to our department between October 2023 and March 2024. Most of these requests (approximately 60%) were for mouthpiece fabrication for tooth movement and prosthetic protection, followed by tartar deposition and poor oral hygiene, and close examination of upset teeth (Figure 2B).

Number and nature of dental problems during general anesthesia

A total of 3.5 incidents per 10,000 cases occurred after the establishment of the clinic, a significant decrease from the number of incidents that occurred before the establishment of the clinic (p=0.036, chi-square test). The dental incidents of chipped, broken, or ripped teeth included the maxillary incisors, mandibular molars, and mandibular incisors. Issues related to teeth condition included tooth loss and prosthetic detachment. There were no differences before and after the opening of the preoperative outpatient clinic. Of the patients experiencing incidents, seven underwent preoperative oral screening, and four (57.1%) of these were identified as having loose teeth. Although the affected patients were advised to undergo dental intervention, they did not consent to it prior to surgery. After the incidents, removal by upper gastrointestinal endoscopy was performed in four patients (12.5%). More than half of the patients reported problems during intubation and extubation of anesthesia, both before and after the opening of the preoperative outpatient clinic (Table 1).

**Table 1:** Number and nature of incidents in general anesthesia (1/2012–12/2024: 32 cases; before opening: 24 cases; after opening: eight cases) NA: not applicable

		Preoperative outpatient clinic	
		Before opening	After opening
		(1/2012-10/2019: 7y 10m)	(11/2019-12/2024: 5y 2m)
1	Number of cases	68,659	52,638
	(Cases/year, per 10,000)	3.5	1.5 p=0.036 (p<0.05; chi-square test)
				2	Parts (cases)		
	Upper front teeth	9	5
	Upper molar teeth	2	0
	Lower front teeth	3	1
	Lower molar teeth	7	1
	Unknown	3	1
	Total	24	8
3	Condition of the teeth (cases)		
	Dropping out	9	5
	Breaking off	5	0
	Prosthesis coming off	9	3
	Unknown	1	0
	Total	24	8
4	Patients in whom oral screening was performed before surgery (7 patients), Cases where the patient was informed of the need for a dental examination owing to the presence of a moving tooth; however, consent was not provided.	NA	4 cases (57.1 %)
5	Cases in which removal was performed using an upper gastrointestinal endoscopy after the occurrence	2 cases	2 cases
	(4 cases (12.5%))		
6	Trouble causes		
	During intubation	8	4
	During extubation	7	2
	Unknown	9	2

Survey of anesthesiologists' awareness of the oral cavity

We received responses from 26 anesthesiologists at our hospital. The respondents included both qualified anesthesiologists and physicians in anesthesiology training, all of whom were engaged in anesthesia management. The number of years of experience of the anesthesiologists ranged from one to 40 years, with 14 having <10 years, four having 11-20 years, and eight having >20 years of experience. Twenty-one (80.8%) physicians felt that oral screening was very useful, and 17 (65.4%) felt that dental problems could be sufficiently resolved through preoperative oral screening. All physicians stated that they were particularly mindful of potential dental-related complications during anesthesia management because of the documented findings of dental hygienists following oral screenings (Table 2).

**Table 2:** Survey of anesthesiologists' awareness of the oral cavity (experience: 1–40 years; number of responses: 26)

	Questions	Answers	
1	Do you feel that oral screenings by dental hygienists are useful?	□ Very useful	21 (80.8%)
		□ Useful to some extent	5 (19.2%)
		□ Somewhat useful	0 (0%)
		□ Minimally useful	0 (0%)
2	Have any dental problems been resolved through preoperative oral screening?	□ Yes, very much so	17 (65.4%)
		□ Yes, some resolution	9 (34.6%)
		□ Yes, somewhat	0 (0%)
		□ Not really	0 (0%)
3	Are you able to be mindful of tooth fractures and dislodging during anesthesia management based on the documentation of the oral screening by a dental hygienist?	□ Yes, this has been very helpful	26(100%)
		□ This has been somewhat helpful	0 (0%)
		□ No, I have not used the documentation preoperatively	0 (0%)

## Discussion

Our preoperative outpatient clinic was established in May 2019, and oral screenings were conducted by dental hygienists in November 2019. At the clinic, a dental hygienist conducts an oral screening of a patient, a nurse assesses the patient’s vital signs, and a pharmacist reviews the patient’s active medications. The final component of the screening clinic includes a preoperative assessment of the patient by an anesthesiologist. Two dental hygienists conduct the oral screenings; the examination takes approximately five minutes, and a total of 36 oral screenings are performed each day. The condition of the mouth, such as the presence of loose teeth and prosthetic devices, is recorded in the patient’s chart using specific symbols, which all staff involved in the treatment can view. If dental problems such as loose teeth and poor oral hygiene are detected before surgery, the patient is referred to the hospital's dental department or to their family dentist for intervention. The number of preoperative outpatient consultations at the hospital is 8,300 per year, and there are approximately 1,200 dentistry referrals annually (15%). These numbers are less than those reported by other medical facilities with dental facilities [5].

Most patients (60%) were referred to our department from the preoperative outpatient clinic for conducting a detailed examination for mouthguard fabrication owing to teeth with slight mobility or prosthetic devices, and other referrals were for patients with poor oral hygiene and patients with significant tooth mobility. Mouthguards are fabricated as needed in a process that involves placing 2 mm of thermoplastic ethylene vinyl acetate onto an intraoral impression model. These are tried on preoperatively, and the patients are instructed to wear the guards when they arrive, before entering the operating theater. The incidence of dental problems during general anesthesia after the establishment of the preoperative outpatient clinic was 0.015% (1.5/10,000 cases), which was significantly lower than that before the clinic was established, and it was also lower than that reported in previous studies [6, 7]. These findings indicate that preoperative oral screenings by dental hygienists are useful and effective. The most common site of dental problems is the maxillary anterior teeth, which is consistent with previous reports [8]. This finding suggests that mouthguards can prevent damage to teeth from laryngoscopes and endotracheal tubes. Our results also suggest that dental problems occur during anesthesia intubation and extubation, which may be prevented by wearing a mouthpiece.

Selective dental intervention through preoperative oral screening has the following advantages. Oral screening can be performed on all patients undergoing anesthesia, eliminating the problem of dental staff shortages. Screenings can alert anesthesiologists to possible dental problems that may occur during intubation and extubation, and such problems can be subsequently avoided through cooperation between medical and dental staff. Patients are informed of their dental problems before surgery, resulting in fewer postoperative problems. In the questionnaire survey, anesthesiologists also indicated that oral screenings performed by dental hygienists before surgery are useful. However, our oral screening system does have challenges. The mouth can be examined, but detailed examinations of lesions in the bone cannot be performed. Imaging tests can help detect lesions that cannot be identified by visual or clinical examination alone [9]. In addition, we are unable to provide postoperative oral management. Dental infections during the perioperative period have been reported to occur at a frequency of 8.2% [10]. Funahara et al. found that the bacteria levels in saliva increase after surgery. When patients are fasting (as may be required postoperatively), the self-cleansing action of the mouth decreases, and the salivary bacterial level increases to approximately 10 times that before surgery. These changes in bacterial levels are comparatively lower in patients with oral intake [11, 12]. We believe that oral management is critical before and after surgery. Moreover, postoperative oral management should involve collaboration with local dental clinics where patients receive dental examinations and care before surgery.

Moreover, this study has several notable things. First, it was conducted at a high-volume tertiary hospital, involving approximately 11,000 general anesthesia cases per year, allowing for a large dataset. Second, the study covered a long pre- and post-implementation period, providing a comprehensive evaluation of the impact of the oral screening system. Third, the study design combined objective data (incident reports from anesthesia records) with subjective data (survey of anesthesiologists’ perceptions), which strengthened the overall validity of the findings.

However, this study also has several limitations. It employed a retrospective design, which is subject to underreporting and detection bias regarding dental incidents. The absolute number of events (n = 32) was small, which limits the statistical power to detect small effect sizes. Additionally, temporal confounding factors, such as other patient safety initiatives introduced during the study period, cannot be completely ruled out. Another limitation is the lack of patient-level data (e.g., dental status, age, comorbidities, or type of anesthesia), which may have influenced the risk of dental injury. Furthermore, although dental hygienists identified loose teeth in some patients, noncompliance with preoperative dental treatment advice may have affected outcomes. The absolute risk reduction observed was approximately 3.7 incidents per 10,000 anesthesia cases (95% CI: 0.2-7.2 per 10,000). Future prospective or multicenter studies with controlled confounders are warranted to validate these results.

Despite these limitations, the findings suggest that implementing preoperative oral screening by dental hygienists can meaningfully reduce dental complications during anesthesia and enhance communication between anesthesiologists and dental staff.

## Conclusions

In conclusion, preoperative oral screening by dental hygienists was found to be useful in identifying and preventing potential dental problems that could occur during anesthesia management. Although providing dental interventions for all patients remains challenging due to human resource and time constraints, the screening system effectively contributed to safer anesthesia management by reducing the incidence of dental complications.

## Figures and Tables

**Figure 3:**
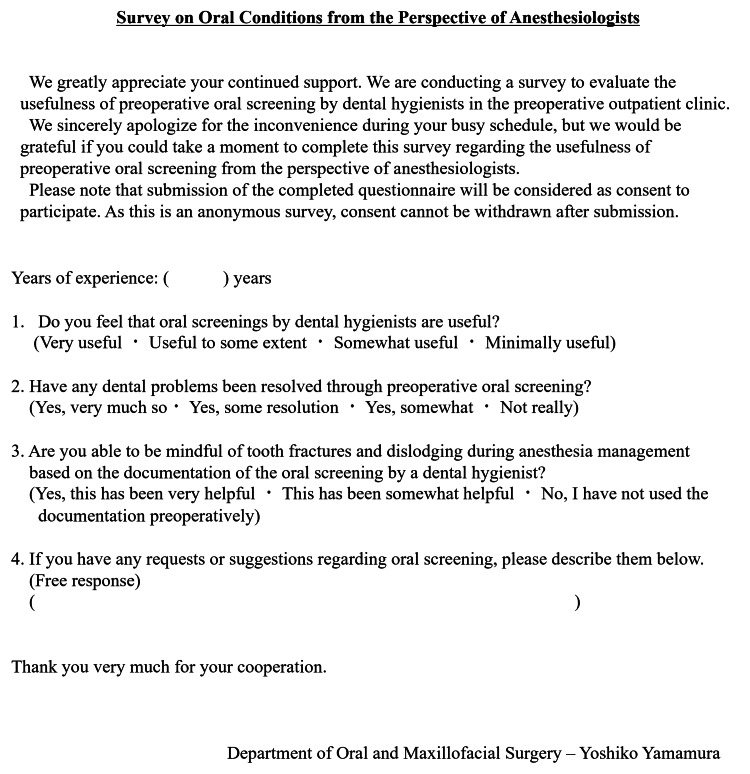
Survey on oral conditions from the perspective of anesthesiologists

## Appendices

Appendix A 
